# Transgenic rescue of phenotypic deficits in a mouse model of alternating hemiplegia of childhood

**DOI:** 10.1007/s10048-015-0461-1

**Published:** 2015-10-13

**Authors:** Greer S. Kirshenbaum, James Dachtler, John C. Roder, Steven J. Clapcote

**Affiliations:** Lunenfeld-Tanenbaum Research Institute, Mount Sinai Hospital, University Avenue, Toronto, ON M5G 1X5 Canada; Institute of Medical Science, University of Toronto, Toronto, ON M5S 1A8 Canada; School of Biomedical Sciences, Garstang Building, University of Leeds, Leeds, LS2 9JT UK

**Keywords:** Alternating hemiplegia, Transgenic rescue, Na^+^,K^+^-ATPase α3, *Atp1a3*, Mice

## Abstract

Missense mutations in *ATP1A3* encoding Na^+^,K^+^-ATPase α3 are the primary cause of alternating hemiplegia of childhood (AHC). Most *ATP1A3* mutations in AHC lie within a cluster in or near transmembrane α-helix TM6, including I810N that is also found in the *Myshkin* mouse model of AHC. These mutations all substantially reduce Na^+^,K^+^-ATPase α3 activity. Herein, we show that *Myshkin* mice carrying a wild-type *Atp1a3* transgene that confers a 16 % increase in brain-specific total Na^+^,K^+^-ATPase activity show significant phenotypic improvements compared with non-transgenic *Myshkin* mice. Interventions to increase the activity of wild-type Na^+^,K^+^-ATPase α3 in AHC patients should be investigated further.

**Electronic supplementary material:**

The online version of this article (doi:10.1007/s10048-015-0461-1) contains supplementary material, which is available to authorized users.

## Introduction

Alternating hemiplegia of childhood (AHC; OMIM: 614820) is a rare neurodevelopmental disorder which manifests as episodic hemiplegia starting in the first 18 months of life, with a spectrum of persistent motor, movement, and cognitive deficits that become progressively more apparent with age [1, 2]. Approximately half of AHC patients present with epilepsy; the seizures are predominantly partial but may manifest as status epilepticus requiring urgent medical attention [2]. Heterozygous missense mutations of the *ATP1A3* gene, encoding the Na^+^,K^+^-ATPase α3 subunit, have been identified as the primary cause of AHC [3]. Belonging to the type II P-type ATPase family of proteins that have a transmembrane domain permeable to specific ions, Na^+^,K^+^-ATPases are membrane-bound transporters that harness the energy of ATP hydrolysis to pump three Na^+^ ions out of the cell in exchange for two K^+^ ions moving inward [4]. The α3 subunit has ten transmembrane α-helices which contain the Na^+^- and K^+^-binding sites and the cytoplasmic domains involved in ATP hydrolysis [4]. Most *ATP1A3* mutations in AHC patients lie within a cluster in or near transmembrane α-helix TM6 [5], including three affecting the isoleucine at position 810: I810F [5], I810N [6], and I810S [7]. Flunarizine, a non-selective calcium entry blocker targeting voltage-dependent calcium channels, reduces the severity, duration, or frequency of hemiplegic attacks in some patients [6], while two AHC patients—positive for T804I and D923N, respectively—have shown marked improvements in symptoms when treated with a ketogenic diet [8, 9]. However, the complexity and severity of AHC make it imperative that new therapeutic options specifically targeting Na^+^,K^+^-ATPase α3 be explored.

To better understand the consequences of Na^+^,K^+^-ATPase α3 dysfunction and explore potential therapeutic approaches for this disorder, investigations in animal models harboring the same mutations as human patients are needed. Heterozygous *Myshkin* (*Atp1a3*^*Myk*/+^; *Myk*/+) mutant mice have an I810N amino acid substitution in transmembrane α-helix TM6 identical to that present in AHC [6, 10]. A 12-year-old Chinese male with the I810N mutation is reported to have AHC with developmental delay and epilepsy [6]. *Myk*/+ mice often show hypokinesia upon arousal and move with a paretic, tremulous gait that becomes transiently more severe after stress (Supplementary Video 1 and 2) [11]. *Myk*/+ mice have not, however, been observed by video monitoring over 24 h, so it is not known whether they exhibit frank hemiparesis or hemiplegia when left undisturbed in the home cage. Other phenotypic abnormalities include decreased body mass, neuronal hyperexcitability, increased susceptibility to epileptic seizures, motor dysfunction, and cognitive impairment [10–12]. Molecular modeling of I810N has shown that it brings about severe structural effects on Na^+^,K^+^-ATPase α3, including the capacity for efficient K^+^ movement along the K^+^ access pathway [11].

Missense mutations in AHC patients and *Myk*/+ mice all substantially reduce Na^+^,K^+^-ATPase α3 activity [8, 13]. I810N in *Myk*/+ mice is functionally a null allele of *Atp1a3* that encodes a normally expressed, but catalytically inactive, Na^+^,K^+^-ATPase α3 enzyme [8]. Brain-specific total Na^+^,K^+^-ATPase activity (contributed by α1, α2, and α3 isoforms) is 64 % of wild-type levels in *Myk*/+ mice [14], but 84 % of wild-type levels in heterozygous *Atp1a3*^tm1Ling/+^ mice that have a point mutation in intron 4 of the *Atp1a3* gene [14, 15]. Since *Atp1a3*^tm1Ling/+^ mice do not have visible neurological defects nor restricted growth [15], we hypothesized that increasing brain Na^+^,K^+^-ATPase activity from 64 % to closer to 84 % of wild-type levels would mitigate disease phenotype severity in *Myk*/+ mice.

Transgenic (Tg)-*Atp1a3*^1Stcl^ mice carry a bacterial artificial chromosome (BAC) transgene of *Mus musculus molossinus* origin (MSMg01-344N17) that contains the wild-type *Atp1a3* gene and its promoter [10]. The Tg-*Atp1a3*^1Stcl^ transgene was previously shown to increase Na^+^,K^+^-ATPase α3 protein expression by 58 % and brain Na^+^,K^+^-ATPase activity from 64 to 80 % of wild-type levels in *Myk*/+ mice [10]. The purpose of the present study was to determine whether this increase in brain Na^+^,K^+^-ATPase activity, to a level comparable with that of *Atp1a3*^tm1Ling/+^ mice (~80 % of wild-type), would have remedial effects in phenotypic tests in which *Myk*/+ mice show clear deficiencies.

## Materials and methods

### Subjects

*Myk*/+ mice were genotyped by the presence of an *Eco*O109I (New England BioLabs) restriction site using PCR primers F, 5′-CTG CCG GAA ATA CAA TAC TGA-3′ and R, 5′-ATA AAT ACC CCA CCA CTG AGC-3′. Hemizygous Tg-*Atp1a3*^1Stcl/+^ (Tg/+) mice were genotyped using PCR primers F, 5′-TGA CAT TGT AGG ACT ATA TTG C-3′ and R, 5′-GTT AAA GGT GTG AGG CAC AGA-3′ spanning the T7 vector-insert boundary. Both lines were backcrossed for 11 (Tg/+) or 21 generations (*Myk*/+) to the C57BL/6NCr strain (NCI-Frederick). *Myk*/+ males were crossed with Tg/+ females to yield wild-type (+/+), *Myk*/+, Tg/+, and *Myk*/+/Tg (I810N + Tg-*Atp1a3*^1Stcl^) littermates. Mice were weaned at 4 weeks of age and grouped housed (two to five mice/cage) with same-sex littermates. Supplementary food pellets were provided on the cage floor for the first 2 weeks post-weaning because of the small size of *Myk*/+ mice. Mice were weighed at 4 and 8 weeks of age using a Scout Pro portable balance (Ohaus).

### Behavioral procedures

*Myk*/+, +/+, Tg/+, and *Myk*/+/Tg littermates (*n* = 12/genotype) were tested at 8–14 weeks of age. Males and females were included in balanced numbers. Subjects were handled daily for 5 min/day for 7 days prior to behavioral testing, which was conducted during the light phase (0900–1700 h). Prior to experiments, subjects were left undisturbed in the testing environment for 30 min to allow for acclimation. A solution of 70 % ethanol was used to clean surfaces and equipment between subjects. The order of tests, with a rest period of 3–5 days between each test, was as follows: body weight → balance beam → Morris water maze → fear conditioning.

### Balance beam

Mice were given five training trials on a 90-cm-long, 18-mm-wide beam elevated 50 cm above a padded base. A 60-W lamp at the start platform served as an aversive stimulus, whereas the opposite end of the beam entered a darkened escape box baited with food pellets. Performance on the beam was quantified in a test trial given 24 h after training by measuring the time that it took for the mouse to traverse the beam and the number of hind foot slips that occurred in the process.

### Visible platform water maze

The water maze consisted of a cylindrical tub of ivory-colored acrylic sheet (117-cm diameter; 30-cm depth) that was filled with water (26 ± 1 °C temperature) to 11 cm below the rim. A circular platform (10-cm diameter) made of transparent acrylic sheet was submerged 1 cm below the water surface at the center of the pool. The platform location was indicated by a high-contrast striped marker rising 13 cm above the water surface. Each subject was given four training trials. At the start of each trial, the mouse was placed by the tail into the water, immediately facing the perimeter, at one of the cardinal compass points (north, south, east, or west), and then was allowed a maximal time of 90 s to locate the platform. Finding the platform was defined as staying on it for at least 2 s. A closed-circuit television camera was mounted onto the ceiling directly above the center of the pool to convey subject swimming trajectories and parameters to an electronic image analyzer (HVS Image), which extracted and stored the X-Y coordinates of the subject’s position at sample points every 0.01 s. Behavioral variables were quantified with the aid of HVS Water 2020 (HVS Image).

### Contextual fear conditioning

Experiments were conducted in a fear-conditioning chamber (MED Associates; 25-cm height × 30-cm width × 25-cm length) with a removable grid floor of 36 stainless steel rods (3.2-mm diameter, 4.7 mm apart) connected to a constant current shock generator. FreezeFrame 1.6e software (Actimetrics) administered foot shocks, recorded video images of the chamber, and monitored the activity of subjects throughout the procedure. For the training phase, mice were placed in the chamber for 2 min 28 s, after which, they received a continuous scrambled foot shock of 0.75 mA for 2 s and then remained in the chamber for an additional 30 s before being returned to their home cage. Twenty-four hours following training, mice were returned to the fear-conditioning chamber to evaluate their contextual fear memory. Freezing to the context was recorded at 0.25-s intervals using FreezeFrame for 3 min, and then, mice were returned to their home cage.

### Statistical analysis

All statistics were calculated by STATISTICA (StatSoft). Data were subjected to analysis of variance (ANOVA) with *Atp1a3* genotype, Tg genotype, and sex as between-subjects factors. No sex by *Atp1a3* genotype or sex by Tg genotype interaction was observed for any variable measured. When ANOVA detected statistically significant main effects, pairwise differences were evaluated using Tukey-Kramer post hoc multiple comparison tests, with significance set at *p* < 0.05. Student’s paired *t* test was used to compare baseline freezing with contextual freezing. All values reported in the figures are expressed as mean ± standard error of the mean (SEM).

## Results and discussion

### Body weight

**Fig. 1:**
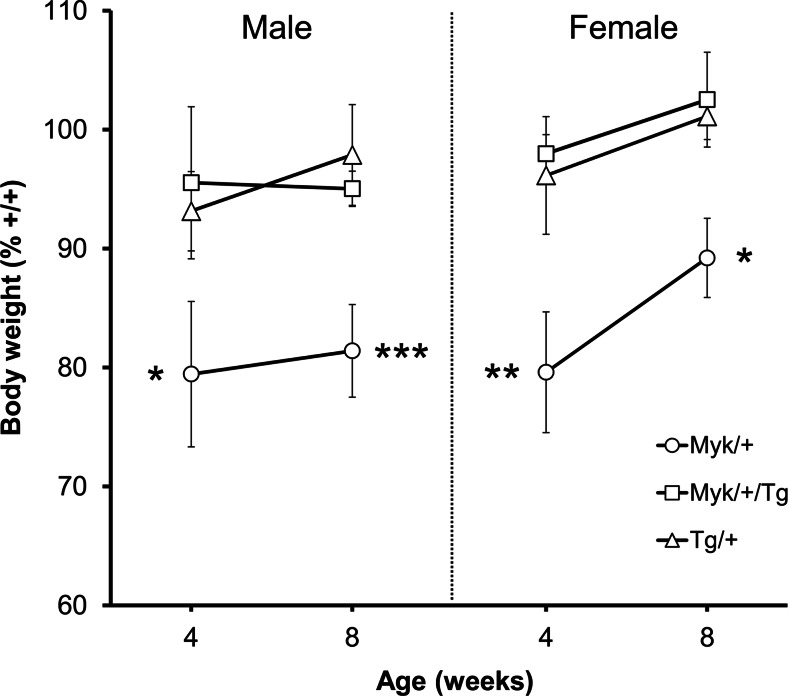
Body weight (% of +/+; mean ± SEM) of male (*n* = 6/genotype) and female (*n* = 6/genotype) *Myk*/+, *Myk*/+/Tg, and Tg/+ mice at 4 and 8 weeks of age. For body weight at 4 weeks, main effects of *Atp1a3* genotype (*F*(1, 42) = 8.41, *p* < 0.01), sex (*F*(1, 42) = 7.40, *p* < 0.01), and *Atp1a3* genotype by Tg genotype interaction (*F*(1, 42) = 12.69, *p* < 0.001) were observed. For body weight at 8 weeks, main effects of *Atp1a3* genotype (*F*(1, 42) = 14.18, *p* < 0.001), Tg genotype (*F*(1, 42) = 8.84, *p* < 0.01), sex (*F*(1, 42) = 82.72, *p* < 0.0001), and *Atp1a3* genotype by Tg genotype interaction (*F*(1, 42) = 10.83, *p* < 0.01) were observed. *p < 0.05; **p < 0.01; ***p < 0.001 compared with +/+ mice

AHC patients tend to be of short stature and low weight [2, 16]. We measured the body weight of mice aged 4 weeks, when they were weaned, and aged 8 weeks, when behavioral testing commenced. At both ages, the body weight of *Myk*/+ mice was significantly less than that of the other genotypes (Fig. 1). Relative to +/+ mice, the 4-week weight of *Myk*/+ mice was less by 20.4 ± 5.1 % in females and by 20.6 ± 6.1 % in males. By 8 weeks, body weight was less by 10.8 ± 3.3 % in *Myk*/+ females and by 18.6 ± 3.3 % in *Myk*/+ males, and a sex by *Atp1a3* genotype interaction approaching significance (*F*(1, 42) = 3.66, *p* = 0.0626) was observed. *Myk*/+/Tg mice did not show any significant deficits in body weight, as confirmed by the significant *Atp1a3* genotype by Tg genotype interactions observed (Fig. 1). In a previous study, we found that the 9-week weight of *Myk*/+ mice was less by 18.8 % in males and 16.1 % in females [10]. The smaller deficit of adult *Myk*/+ females in the present study could reflect a sex-specific response to differing husbandry conditions. Male heterozygous *Atp1a3*^D801N/+^ mice, which carry the most common mutation causing AHC (D801N), also show lower body weight at 8 and 9 weeks of age, but *Atp1a3*^D801N^ genotype had no effect on the body weight of female mice at any age [17].

### Balance beam

**Fig. 2:**
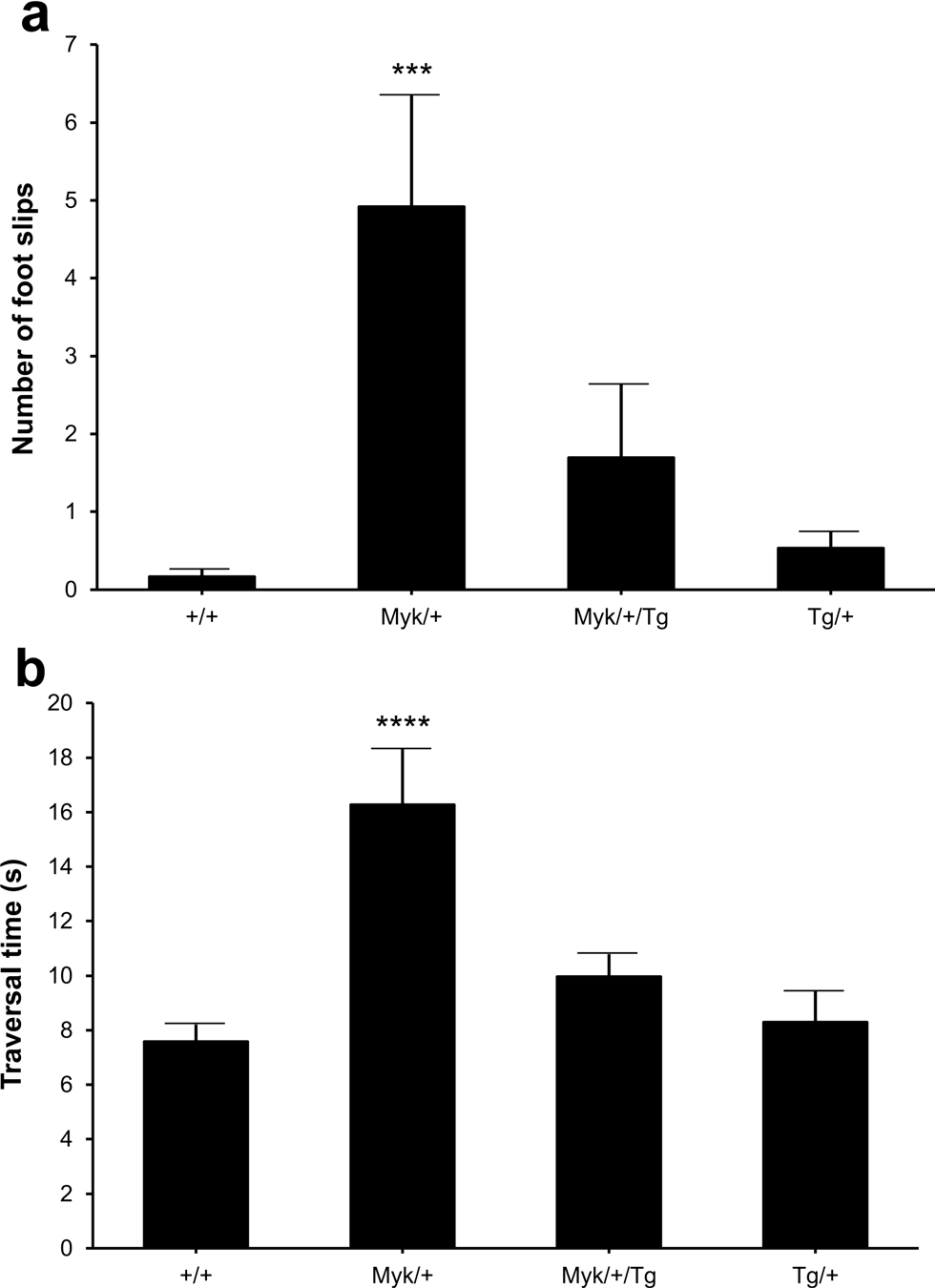
Balance beam performance of +/+, *Myk*/+, *Myk*/+/Tg, and Tg/+ mice (*n* = 12/genotype). **a** Number of hind foot slips (mean ± SEM) and **b** traversal time (s; mean ± SEM) when traversing a narrow beam. For the number of foot slips, main effects of *Atp1a3* genotype (*F*(1, 42) = 14.11, *p* < 0.001) and *Atp1a3* genotype by Tg genotype interaction (*F*(1, 42) = 5.02, *p* < 0.05) were observed. For traversal time, main effects of *Atp1a3* genotype (*F*(1, 42) = 22.19, *p* < 0.0001), Tg genotype (*F*(1, 42) = 5.52, *p* < 0.05), and *Atp1a3* genotype by Tg genotype interaction (*F*(1, 42) = 9.54, *p* < 0.01) were observed. ***p < 0.001; ****p < 0.0001 compared with +/+ mice

Abnormalities of fine motor skills are present in the vast majority of AHC patients and become more evident with age [2, 18]. As a measure of motor coordination, we tested mice on a balance beam, which is useful for detecting subtle deficits in motor skills and balance that may not be detected by other motor tests, such as the rotarod [19]. At 24 h after training, *Myk*/+ mice showed a significantly greater number of hind foot slips than the other genotypes as they traversed the beam (Fig. 2a). *Myk*/+ mice also took longer than the other genotypes to traverse the beam (Fig. 2b). By contrast, the number of foot slips and the traversal time of *Myk*/+/Tg mice were not significantly different from those of +/+ mice, as confirmed by the significant *Atp1a3* genotype by Tg genotype interactions observed (Fig. 2). *Atp1a3*^D801N/+^ mice have also performed poorly in the balance beam test [17].

### Visible platform water maze

Cognitive impairment has been observed in 94 % [2] and 100 % [1] of different AHC patient cohorts studied. The visible platform version of the water maze is a simple associative non-spatial task believed to be independent of hippocampal function [20]. This procedure introduces the mice to the main task requirements (e.g., swimming, the fact that the platform is not near the perimeter, and climbing on the platform to escape) and assesses whether they have the performance skills necessary for spatial memory to be assessed in the hidden platform task. In this rather stressful condition, mice are required to maintain sufficient behavioral flexibility to overcome their initial tendency to swim along the wall of the pool, an innate behavior called wall hugging or thigmotaxis [20]. Then, they must learn that they are returned to their warm home cage if they climb and stay on the escape platform indicated by a marker rising above the water.

**Fig. 3:**
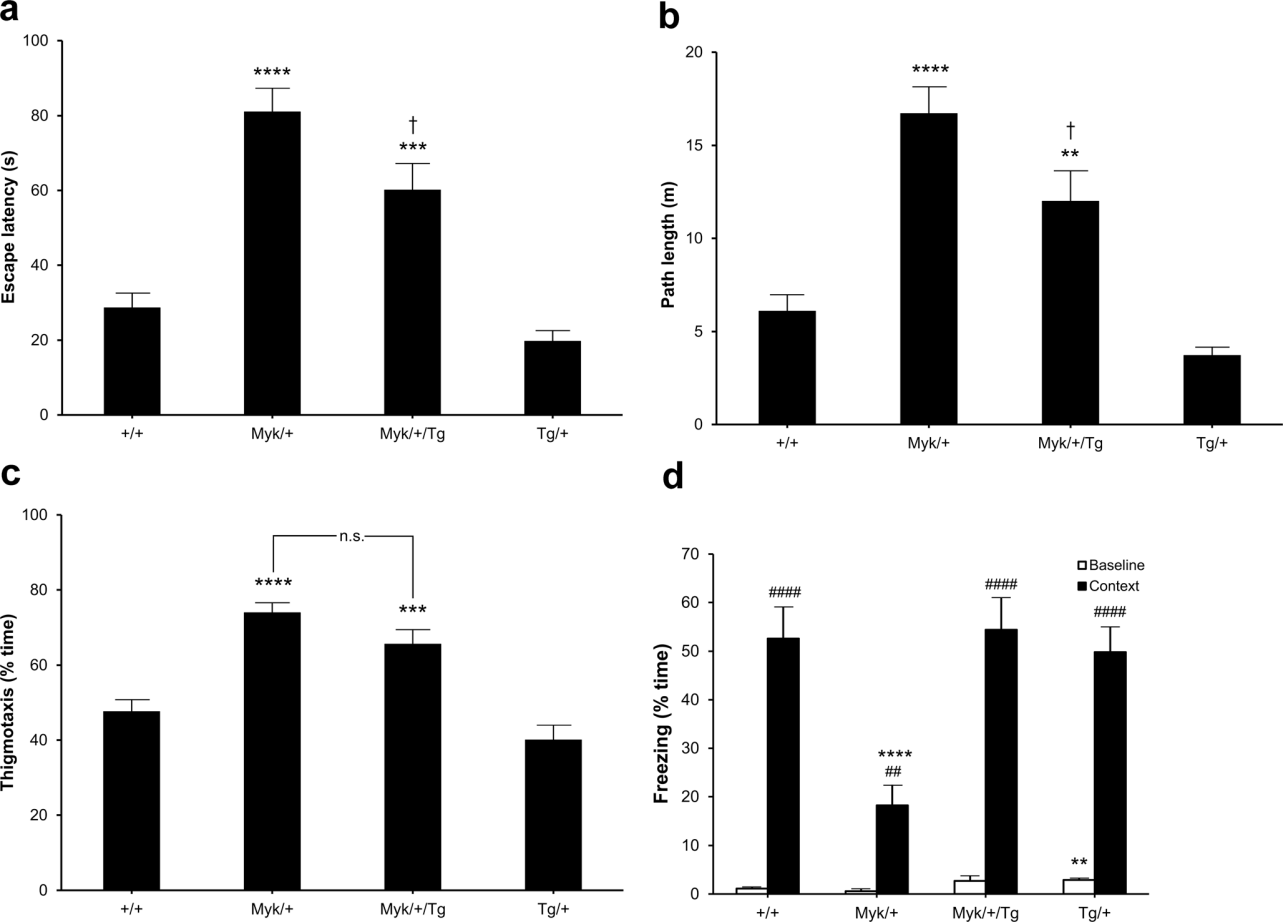
Water maze and fear-conditioning performance of +/+, *Myk*/+, *Myk*/+/Tg, and Tg/+ mice (*n* = 12/genotype). **a** Escape latency (s; mean ± SEM). Main effects of *Atp1a3* genotype (*F*(1, 42) = 77.09, *p* < 0.0001) and Tg genotype (*F*(1, 42) = 7.79, *p* < 0.01) were observed for latency. **b** Swim path length (m; mean ± SEM). Main effects of *Atp1a3* genotype (*F*(1, 42) = 67.20, *p* < 0.0001) and Tg genotype (*F*(1, 42) = 9.10, *p* < 0.01) were observed for path length. **c** Thigmotaxis (% time; mean ± SEM). Main effects of *Atp1a3* genotype (*F*(1, 42) = 53.10, *p* < 0.0001) and Tg genotype (*F*(1, 42) = 4.59, *p* < 0.05) were observed for thigmotaxis. **d** Freezing levels (% time; mean ± SEM) of mice when test-naïve (baseline) and when returned to the chamber 24 h after training (context). For baseline freezing, a main effect of Tg genotype (*F*(1, 42) = 11.60, *p* < 0.01) was observed. For context freezing, main effects of *Atp1a3* genotype (*F*(1, 42) = 5.33, *p* < 0.05), Tg genotype (*F*(1, 42) = 7.40, *p* < 0.01), and *Atp1a3* genotype by Tg genotype interaction (*F*(1, 42) = 10.01, *p* < 0.01) were observed. **p < 0.01; ***p < 0.001; ****p < 0.0001 compared with +/+ mice. †*p* < 0.05 compared with *Myk*/+ mice. ##*p* < 0.01; ####*p* < 0.0001 compared with baseline freezing for each genotype. *n.s.* not significant

Over four trials, only +/+ and Tg/+ mice showed quick orientation toward the visually marked platform location. *Myk*/+ and *Myk*/+/Tg mice took more time (Fig. 3a) and swam further (Fig. 3b) before reaching the visible platform. *Myk*/+/Tg mice performed marginally better than *Myk*/+ mice, but the overall performance of both was poor, as confirmed by the lack of *Atp1a3* genotype by Tg genotype interactions for latency and path length. There were no significant differences between genotypes in swim speed and floating. The deficient performances of *Myk*/+ and *Myk*/+/Tg mice were largely due to their excessive thigmotaxis (Fig. 3c), an effect of the I810N mutation not attenuated by the *Atp1a3*^1Stcl^ transgene. The mutation reduced behavioral flexibility to such a degree that *Myk*/+ and *Myk*/+/Tg mice were practically unable to overcome thigmotaxis, as previously observed in mice with forebrain-specific knockout of the TrkB receptor [22]. Excessive thigmotaxis has also been observed in mice with retinal degeneration [23], but the normal head tracking of *Myk*/+ mice in an optokinetic drum suggests that their vision is not impaired [14].

*Atp1a3*^D801N/+^ mice have also performed poorly in the visible platform task [17]. *Atp1a3*^tm1Ling/+^ mice were given four trials per day for 6 days, in which the visible platform position was changed at random on each trial. *Atp1a3*^tm1Ling/+^ mice took significantly longer to reach the visible platform on days 2–5 but caught up to wild-type controls by day 6 [15].

### Contextual fear conditioning

Contextual fear conditioning assesses a memory for the association between an aversive stimulus, such as a mild foot shock, and a salient environmental cue, the test chamber (“context”). Rodents with good memory have a natural tendency to freeze (suppress all movement) upon re-presentation of the context. Contextual fear conditioning is dependent upon the integrity of the hippocampus [24]. Baseline freezing, before the administration of a foot shock, was less than 3 % for all genotypes, although the baseline freezing of Tg/+ mice was significantly greater than that of +/+ and *Myk*/+ mice (Fig. 3d). At 24 h after receiving the foot shock, all genotypes increased their level of freezing compared with baseline, but *Myk*/+ mice showed a significantly lower level of contextual freezing than the other genotypes. By contrast, *Myk*/+/Tg mice did not show any deficit in contextual fear conditioning, as confirmed by the significant *Atp1a3* genotype by Tg genotype interaction observed (Fig. 3d). Unlike *Myk*/+ mice, *Atp1a3*^D801N/+^ mice have not shown any deficit in the formation of fear-related memories in the contextual fear-conditioning paradigm [17].

The clinical severity of AHC is extremely variable, but the rarity of the disorder has made it difficult to study genotype-phenotype correlations. Nevertheless, the high frequency of D801N and E815K, responsible for most AHC cases, has allowed two independent studies to show that E815K causes a more severe phenotype than D801N with respect to age of onset, motor impairment, and a prevalence of status epilepticus [25, 26]. Differences in mutant enzyme activity cannot account for the disease severity associated with E815K, since all of the AHC mutations that have been studied to date—S137Y, I274N, D801N, I810N, E815K, and G947R—were found to result in a catalytically inactive α3 enzyme [10, 13]. However, the recent finding that E815K, but not D801N and G947R, impairs the passive influx of protons into the cell [27] suggests that loss of proton transport is a correlate of severe AHC [28].

The isoleucine at position 810 is recurrently mutated in AHC [5–7], but I810N is one of the rarer mutations, having been found in only two cases to date: a 22-year-old male from Belgium with clinical features of episodic hemiplegia and dystonia triggered by emotional stress, mild ataxia with unstable gait, moderate intellectual disability and autism, and a clinical history of epilepsy with some episodes of status epilepticus [29]; and a 12-year-old Chinese male with clinical features of episodic hemiplegia, quadriplegia, abnormal eye movement, dystonia, epilepsy, and developmental delay [6].

Haploinsufficiency of *ATP1A2* encoding the Na^+^,K^+^-ATPase α2 subunit is associated with familial hemiplegic migraine type 2 [30], but recent functional analysis of α3 subunits suggests that an additional dominant-negative mechanism would contribute to loss of function in AHC [28]. Co-expression of wild-type and mutant α3 subunits (D801N, E815K, and G947R) in *Xenopus laevis* oocytes showed that mutant α3 subunits inhibit wild-type α3 function [28]. This dominant-negative effect may explain why the Tg-*Atp1a3*^1Stcl^ transgene increased brain Na^+^,K^+^-ATPase activity in *Myk*/+ mice by only 16 % [10]. Nevertheless, we have found that this modest increase in brain Na^+^,K^+^-ATPase activity was sufficient to rescue the phenotypic deficiencies of *Myk*/+ mice in body weight, motor coordination, and contextual fear conditioning. Increasing brain Na^+^,K^+^-ATPase activity to 80 % of wild-type levels did not, however, rescue the deficient performance of *Myk*/+ mice in the visible platform version of the water maze, which is consistent with the deficient visible platform performance of *Atp1a3*^tm1Ling/+^ mice whose brain Na^+^,K^+^-ATPase activity is at 84 % of wild-type levels [14, 15]. In conclusion, our results show that a relatively small increase in Na^+^,K^+^-ATPase α3 activity has therapeutic effects in a mouse model of AHC. In light of these findings, interventions to increase the activity of wild-type Na^+^,K^+^-ATPase α3 in AHC patients should be investigated further.

## Electronic supplementary material

Supplementary Video 1 Hypokinesia of *Myk*/+ mouse (center) upon arousal from a resting state. (MP4; 25,215 KB) (MP4 25214 kb)

Supplementary Video 2 Gait of *Myk*/+ mouse before (baseline) and after a 1-min immersion in cold water (7 °C). (MP4; 19,905 KB) (MP4 19904 kb)
